# An Improved Residual Network for Pork Freshness Detection Using Near-Infrared Spectroscopy

**DOI:** 10.3390/e23101293

**Published:** 2021-09-30

**Authors:** Liang Zou, Weinan Liu, Meng Lei, Xinhui Yu

**Affiliations:** 1 School of Information and Electrical Control Engineering, China University of Mining and Technology, Xuzhou 221116, China; liangzou@cumt.edu.cn (L.Z.); weinanliu@cumt.edu.cn (W.L.); lmsiee@cumt.edu.cn (M.L.); 2 Department of Electrical and Computer Engineering, The University of British Columbia, Vancouver, BC V6T 1Z4, Canada

**Keywords:** pork freshness, near-infrared spectroscopy, residual network, squeeze-and-excitation block, deep learning

## Abstract

Effective and rapid assessment of pork freshness is significant for monitoring pork quality. However, a traditional sensory evaluation method is subjective and physicochemical analysis is time-consuming. In this study, the near-infrared spectroscopy (NIRS) technique, a fast and non-destructive analysis method, is employed to determine pork freshness. Considering that commonly used statistical modeling methods require preprocessing data for satisfactory performance, this paper presents a one-dimensional squeeze-and-excitation residual network (1D-SE-ResNet) to construct the complex relationship between pork freshness and NIRS. The developed model enhances the one-dimensional residual network (1D-ResNet) with squeeze-and-excitation (SE) blocks. As a deep learning model, the proposed method is capable of extracting features from the input spectra automatically and can be used as an end-to-end model to simplify the modeling process. A comparison between the proposed method and five popular classification models indicates that the 1D-SE-ResNet achieves the best performance, with a classification accuracy of 93.72%. The research demonstrates that the NIRS analysis technique based on deep learning provides a promising tool for pork freshness detection and therefore is helpful for ensuring food safety.

## 1. Introduction

Pork is one of the most popular meat products in people’s daily diet because it tastes delicious and contains abundant protein, fat, vitamins, as well as other nutrients [1]. Besides providing energy for human, these nutrients also allow for microbial growth and reproduction, which makes pork meat deteriorate easily [2]. Hence, plenty of measures have been taken to keep pork fresh and to extend the shelf life, such as cold storage [3] and cold chain transportation [4]. However, in view of the cost and consumption habits, these effective preservation techniques have not been universally applied and hot, fresh meat still has a high market occupancy in some developing countries. For instance, hot, fresh meat accounts for 60% of market share in China [5], the world’s largest pork producing and consuming country. Hot and fresh pork, preserved without any low temperature treatments, is more vulnerable to spoilage compared with chilled and fresh, or frozen pork. Generally, pork meat is less fresh and smells acidic after 24 h of storage at normal temperature (20 °C) [6]. To protect consumers’ interests and to promote fair competition in markets, it is essential to monitor pork freshness.

Traditional methods for detecting pork freshness mainly include sensory evaluation [7], microbiological testing [8], and physicochemical analysis [9]. Sensory evaluation requires inspectors to determine pork freshness based on color, smell, and other sensory information. This method is easy to used but has strong subjectivity as the evaluation results are susceptible to the inspector’s mood and physical condition. Microbiological testing and physicochemical analysis can accurately determine pork freshness by detecting microbial or physicochemical indexes such as colonies number, total volatile basic-nitrogen (TVB-N), pH, and K value, but they are destructive, time-consuming, and incompatible with the development of the modern meat industry [1].

The near-infrared (NIR) region covers wavelengths from 780 to 2500 nm, which is consistent with the overtone and combination band of hydrogen-containing groups (O–H, C–H, and N–H) [10,11]. As a rapid and nondestructive analysis technique [12,13], NIRS has been widely applied to explore the inner information of samples. For instance, Li et al. [14] proposed an improved Complete Ensemble Empirical Mode Decomposition with Adaptive Noise (CEEMDAN) algorithm to pretreat the NIR spectra of glucose solution. The experimental results show that the developed algorithm combined with permutation entropy can effectively remove noise and select characteristic wavelengths in detecting glucose concentration based on NIRS. Lei et al. [15] enhanced the random forest model with a synthetic minority oversampling technique to analyze the NIR spectral information of coal to obtain its geographic origin and the prediction accuracy reached 97.92%. To rapidly acquire the moisture and amylose content of cereal, Le et al. [16] applied the stacked sparse autoencoder method to extract features of NIR spectral data and verified its effectiveness on corn and rice datasets.

NIRS, combined with various machine learning methods, has been widely used in meat freshness detection as well. For instance, Zhou et al. [17] adopted the NIR spectra in the range of 1000–1799 nm to determine the freshness of bighead carps. In order to predict the TVB-N content of these bighead carps, they proposed an improved partial least-squares regression (PLSR) model based on competitive adaptive reweighted sampling algorithm. To rapidly evaluate pork freshness, Qu et al. [18] proposed a multi-index statistical information fusion (MISF) modeling method based on NIRS. The prediction root mean square error (RMSEP) of the MISF was 3.91, which indicates that this method could achieve a good performance. Li et al. [19] integrated PLSR with a series of spectral preprocessing methods to measure TVB-N content in crabs and the RMSEP of the employed model achieved 3.00. The abovementioned research demonstrates that NIRS can be utilized as a promising tool in meat freshness determination. However, current studies are mainly based on conventional models that require various preprocessing methods to remove random noises and uninformative variables. In general, different combinations and orders of the preprocessing techniques will result in different effects [20]. In addition, incorrect use of preprocessing methods will distort the original signal. Therefore, it is difficult and time-consuming to select optimal preprocessing methods prior to model establishment.

In recent years, driven by the development of big data and computational capability, deep learning models represented by convolutional neural network (CNN) has achieved remarkable success in image processing [21,22], natural language processing [23,24], and speech recognition [25,26]. Different from traditional methods, deep learning models are capable of automatically extracting high-level features from the high-dimensional input data through hierarchical structures [27]. Due to this advantage, a few researchers have employed deep learning coupled with NIRS for qualitative or quantitative analysis. For instance, Chen et al. [28] constructed an end-to-end quantitative analysis model based on CNN to predict the content of moisture, oil, protein, and starch value of corn. The proposed CNN model used raw NIR spectra of corn as input data and then output the prediction of target components without any manual feature selection methods. The results indicate that utilizing CNN for NIRS analysis could simplify the procedure of modeling. Similarly, Zhou et al. [29] applied CNN to discriminate the geographical origin of Tetrastigma hemsleyanum according to its NIR spectrum, and the classification accuracy reached 100%. Although increasing the depth of CNN can extract more abstract features and therefore improve the performance, it may suffer from degradation [30]. In order to solve this problem, He et al. [31] proposed a residual network (ResNet) for image recognition and it has been introduced in the NIRS analysis. For example, Jiang et al. [32] used 1D-ResNet to classify the tobacco cultivation regions. The model outperformed the CNN model (93.16%) and achieved an accuracy of 97.01%. Additionally, Huang et al. [33] built a qualitative analysis model based on ResNet to establish the relationship between NIR spectral vectors and the ingredient contents of medical fungi.

SE block [34] is a channel attention mechanism and can be embedded in existing CNNs to improve model performance. The effectiveness of SE block has been verified in congestive heart failure detection [35] and object detection tasks [36]. Inspired by these works, this study combines the SE block with a 1D-ResNet to investigate its potential in pork freshness classification based on NIRS. To the best of our knowledge, this is the first attempt to utilize a channel attention mechanism in NIRS analysis. The main contributions of this work are as follows:

(1) This paper presents an end-to-end strategy to determine whether the targeted pork is fresh via NIRS. It extracts deep features automatically from raw data, which not only improves the generalization but also avoids the potential error propagation and information reduction. To the best of our knowledge, no prior work has employed deep learning to determine the pork freshness based on NIRS.

(2) Considering the limited samples, the nested cross-validation is employed to evaluate the model performance, which is able to avoid the information leakage.

(3) This study employs a 1D-SE-ResNet model to find the hidden pattern between the NIRS and pork freshness. In order to increase the sensitivity to informative features, we integrate the SE blocks with residual network. The proposed model outperforms the conventional models in terms of classification accuracy, sensitivity, and specificity.

## 2. Materials and Methods

### 2.1. Samples and NIR Measurement

**Figure 1:**
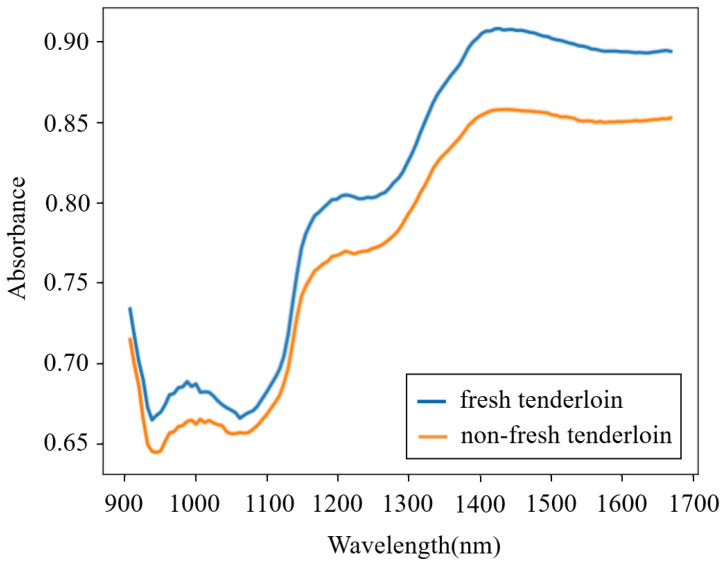
Spectra from a fresh and a non-fresh tenderloin.

Fresh pork samples derived from recently slaughtered pigs were purchased from a local abattoir. Fifteen pieces of pork meat (five pieces each for streaky pork, foreleg muscle, and tenderloin) were bought each day, and this process lasted for 8 days, which in total provided one hundred and twenty fresh samples. To homogenize the meat, each sample was minced by an electric meat grinder and then placed into a round Petri dish. The NIR spectra of all of the fresh samples were obtained using a portable MicroNIR${}^{\mathrm{TM}}$ on-site spectrometer combined with the software MicroNIR${}^{\mathrm{TM}}$ Pro v2.5.1. The spectrometer was set in diffuse-reflection mode, and its wavelength ranged from 908 to 1676 nm. To reduce the random error in thet measurement process, each sample was scanned five times and the average spectrum was adopted. After measurement, the fresh samples were preserved at normal temperature (18–22 °C) in open Petri dishes for 24 h and then were scanned again to acquire the spectra of non-fresh samples. Therefore, a total of 240 spectra were obtained. The spectra from a fresh and a non-fresh tenderloin are shown in Figure 1, which illustrates that the spectra of pork meat with different freshness are similar and that it is difficult for a human to directly determine the pork freshness without the aid of machine learning methods.

### 2.2. Outlier Detection Method

Instrument failures, improper operations, and other factors may cause outliers in the NIRS dataset. An outlier is significantly different from the norm, and its existence interferes with the model performance. Hence, it is essential to detect and exclude outliers before modeling. To address this problem, iteration clipping based on Mahalanobis distance (MD-IC) was employed in this study. Mahalanobis distance (MD) considers the correlations of the variables and is scale-invariant [37]. Before outlier detection, principal component analysis (PCA) was performed to reduce the feature dimension. The 20 principle components that explained more than 99.99% of the variance were retained in consideration of the relatively small size of the dataset. It should be noted that we only adopted the PCA for outlier detection and still used the original spectra as the network’s input. For each sample $x_{i}$ in dataset $X_{m\times n}={\left[x_{1},x_{2},...,x_{m}\right]}^{T}$(*m* is the number of samples, and *n* is the number of wavelength points), the MD between $x_{i}$ and $\bar{x}$ was calculated using Equation (1).

(1) $$D_{M}\left(x_{i},\bar{x}\right)=\sqrt{\left(x_{i}-\bar{x}\right)\Sigma^{-1}{\left(x_{i}-\bar{x}\right)}^{T}}$$

(2) $$\bar{x}=\frac{1}{m}\sum_{p=1}^{m}x_{p}$$

(3) $$\Sigma =\frac{1}{m-1}{\left(X_{m\times n}-\bar{x}\right)}^{T}\left(X_{m\times n}-\bar{x}\right)$$

where $\bar{x}$ is the mean spectrum of samples, *p* denotes the index of sample, and Σ is the corresponding covariance matrix.

The MD-IC detects outliers based on PauTa criterion and selects the 3σ (three times the standard deviation of the MD between each sample and the mean value) as a threshold that is frequently used in NIRS analysis [38]. According to PauTa criterion, outliers might be detected with a confidence probability of 99.7% if the distance between targeted samples and the mean spectrum follows Gaussian distribution. First, the mean value μ and standard deviation σ of the Mahalanobis distances were calculated based on Equations (4) and (5), respectively. Afterwards, each $D_{M}\left(x_{i},\bar{x}\right)\left(i=1,2,3,\ldots ,m\right)$ were examined according to Equation (6). If $|D_{M}\left(x_{i},\bar{x}\right)-\mu |⩾3\sigma$, the sample $x_{i}$ was excluded as an outlier. After outlier exclusion, the MD was recalculated and the above steps were repeated until no outlier was found.

(4) $$\mu =\frac{1}{m}\sum_{p=1}^{m}D_{M}\left(x_{p},\bar{x}\right)$$

(5) $$\sigma =\sqrt{\frac{1}{m}\sum_{p=1}^{m}{\left(D_{M}\left(x_{p},\bar{x}\right)-\mu \right)}^{2}}$$

(6) $$|D_{M}\left(x_{i},\bar{x}\right)-\mu |⩾3\sigma$$

### 2.3. 1D-SE-ResNet Model

#### 2.3.1. Structure of the Model

**Figure 2:**
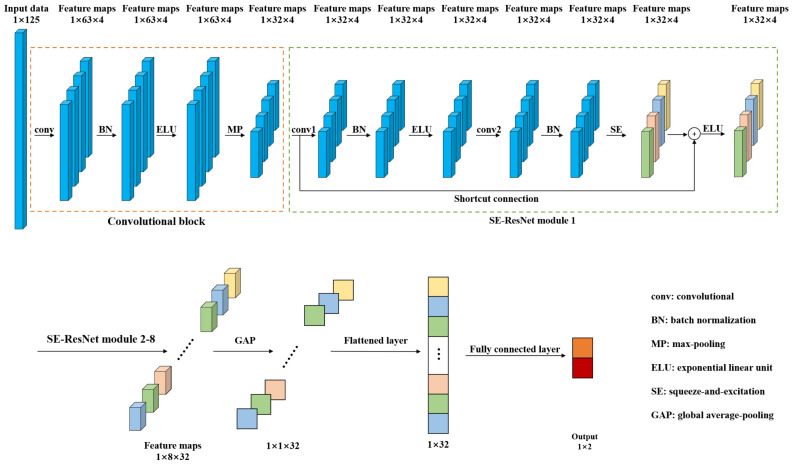
Architecture of the 1D-SE-ResNet.

In order to detect the pork freshness, this paper presents a 1D-SE-ResNet classification model based on NIRS. Compared with traditional models such as SVM and RF, the proposed model, an end-to-end network, can extract features from input data automatically. Figure 2 shows the architecture of 1D-SE-ResNet. It includes a convolutional block, eight SE-ResNet modules, a global average-pooling (GAP) layer, a flattened layer, and a fully connected layer.

Convolutional block and SE-ResNet modules were used to extract features from the input spectra. The convolutional block was composed of a convolutional layer (conv), a batch normalization (BN) layer, an exponential linear unit (ELU) layer, and a max-pooling (MP) layer. The convolutional layer utilizes multiple trainable convolutional kernels to capture different features, and each kernel yields a feature map. Similar to the input spectrum, the kernels are one dimensional as well. BN was adopted to stabilize and accelerate the training process. ELU, a nonlinear function, was used to enhance the expression ability of the model. The MP layer was utilized to reduce the size of the feature map by retaining the salient features. The SE-ResNet module is elaborated on in the following subsection. The GAP was used to average each feature map, and then, these acquired averages were converted into a 1D vector by a flattened layer. The output of the fully connected layer was processed by a softmax function to give a conditional probability for each category. To train the network, the Adam optimizer was adopted and the loss was calculated by cross entropy loss function, which was frequently used in classification tasks [39].

#### 2.3.2. SE-ResNet Module

**Figure 3:**
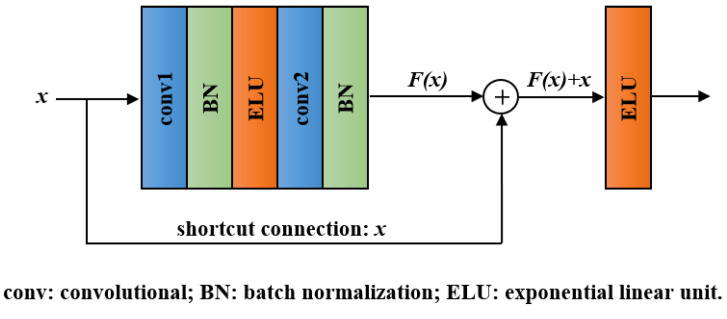
Structure of the residual block.

With the network layers increasing, a degradation problem appeared: accuracy became saturated and then degraded quickly [40]. Residual block, the key module of ResNet, can effectively addresses the degradation problem by introducing a shortcut connection [31]. The structure of a residual block is shown in Figure 3. It involves convolutional layers, BN layers, ELU layers, and a shortcut connection. Except for the shortcut connection, the function of each unit in residual block is the same as that in the convolutional block. In the residual block, we denote the desired underlying mapping as H(x) and let the stacked layers approximate a residual function F(x):=H(x)−x. Hence, the original mapping was recast into F(x)+x. Compared with directly fitting *H*(*x*) using stacked layers, the residual learning is easier to realize and can avoid degradation problem.

**Figure 4:**
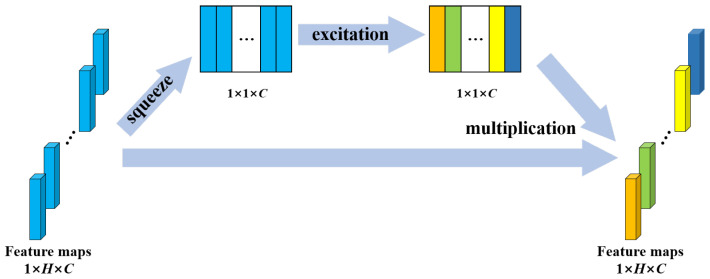
Structure of the SE block. *H* denotes the number of elements in a feature map; *C* denotes the number of feature maps.

Convolutional neural networks extract features by fusing spatial and channel-wise information [41]. SE block is designed to boost the representational power of a model from the aspect of channel relationship. Figure 4 shows the structure of SE block. Multiple feature maps are acquired after convolution operation. However, a few feature maps may carry redundant information. To enhance the informative features and to inhibit the less useful ones, feature recalibration is performed by SE block. First, the squeeze operation implements a global pooling on each feature map and a weight vector is acquired. Then, in excitation operation, fully connection layers and sigmoid activation function are used to redistribute the feature weights. The redistribution is guided by gradient descent algorithm. Finally, the feature maps are reweighted using these weights. In this study, the SE block was placed behind the BN in each residual block to recalibrate the feature maps acquired from the stacked layers. The structure of SE-ResNet module is shown in Figure 2.

#### 2.3.3. Activation Function

As an important unit, the activation function introduces nonlinear factor into the model. A network without activation functions can only realize linear mapping, which is hard to fit nonlinear distributed data. Hence, the activation function plays a significant part in improving the fitting ability of a network. The frequently used activation functions are listed in Table 1.

**Table 1:** Frequently used activation functions.

Activation Function	Equation	
Sigmoid	$\sigma \left(x\right)=\frac{1}{1+e^{-x}}$	(7)
ReLU	$\mathrm{ReLU}\left(x\right)=\max\left(x,0\right)=\left\{\begin{array}{c} x,x\geq 0\\ 0,x<0 \end{array}\right.$	(8)
ELU	$\mathrm{ELU}\left(x\right)=\left\{\begin{array}{c} x,x\geq 0\\ \alpha *(e^{x}-1),x<0 \end{array}\right.$	(9)

ReLU: Rectified Linear Unit; ELU: Exponential Linear Unit; α defaults to 1.0.

### 2.4. Nested Cross-Validation

**Figure 5:**
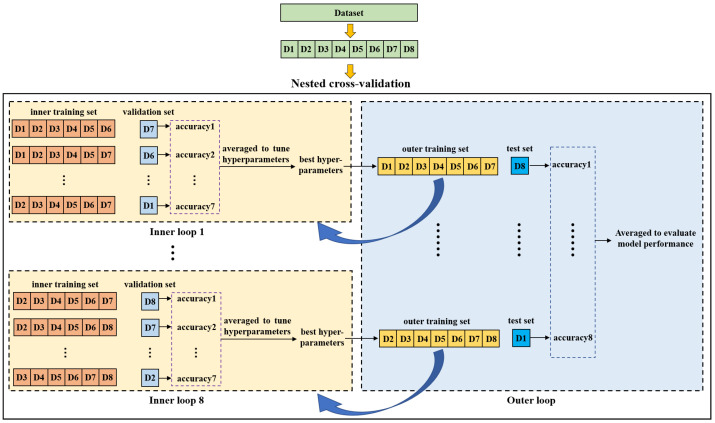
Diagram of the nested cross-validation.

Nested cross-validation is an effective method for estimating the generalization ability of a model and is often used to train a model in which hyperparameters also need to be tuned [42]. Reference [43] demonstrated that the nested cross-validation can give almost unbiased estimation of the true error. Figure 5 shows the diagram of nested cross-validation. It includes inner and outer cross-validation. The purpose of the inner loop is to tune hyperparameters of the model and to choose the optimal ones. The outer loop is used to evaluate the model performance. In this study, we first split the dataset into eight groups (D1-D8) according to the purchase date. Afterwards, one group was selected as a test set while the remaining groups were taken as an outer training set on which a seven-fold cross-validation was performed to search the optimal hyperparameters in the inner loop. For each fold, a group was used as a validation set and the other six groups were used to train the model. The inner cross-validation was performed multiple times to compare different hyperparameters. In each inner cross-validation, the hyperparameters were fixed and evaluated by the average prediction accuracy of the validation sets. The model with the best hyperparameters was trained on the outer training set and then tested on the test set. This process was repeated eight times until all eight groups were tested, and the average indices of test sets were taken as the final results to evaluate the model performance.

### 2.5. Traditional Models Used for Comparison

Support vector machine (SVM), Random Forest (RF), and partial least-squares discrimination analysis (PLS-DA) are frequently used conventional classification models in NIRS analysis. SVM maps the input data into a high-dimensional space through kernel trick and then constructs a hyperplane to separate the samples. In this experiment, the radial basis function (RBF) kernel was employed. The hyperparameters to be tuned in SVM were C (penalty coefficient) and gamma (a parameter of RBF). As an ensemble model, the RF consists of multiple decision trees, the number of which is an important hyperparameter and performs nonlinear modeling. PLS-DA is a linear classification method that combines the PLSR and the discrimination techniques. It utilizes principal components to represent the input spectra and constructs a correlation between these components and the labels. The number of the principal components were determined through the nested cross-validation. To improve the performance of conventional models, three popular preprocessing techniques were adopted, including standardization, smoothing, and PCA. Standardization is a data transformation method that is used to make the input data follow the standard normal distribution. The purpose of smoothing is to reduce the noises in the spectral data but it introduces another hyperparameter (sliding window size). PCA is an unsupervised dimensionality reduction method that aims at extracting features from the input data.

### 2.6. Evaluation Indices of the Model

The performance of the machine learning models used in this study was evaluated by determining the accuracy (*Acc*), precision (*Pre*), sensitivity (*Sen*), and specificity (*Spe*). The parameters were calculated as follows:

(10) $$Acc=\frac{TP+TN}{TP+FN+FP+TN}$$

(11) $$Pre=\frac{TP}{TP+FP}$$

(12) $$Sen=\frac{TP}{TP+FN}$$

(13) $$Spe=\frac{TN}{TN+FP}$$

where *TP* (True Positive) is the number of fresh samples that are correctly classified as fresh, *TN* (True Negative) is the number of non-fresh samples that are correctly classified as non-fresh, *FN* (False Negative) is the number of fresh samples that are wrongly classified as non-fresh, and *FP* (False Positive) is the number of non-fresh samples that are wrongly classified as fresh. These four performance indices are between 0 and 1. The higher the value, the better the classification performance of the corresponding classifier.

## 3. Results and Discussion

### 3.1. Outlier Detection

**Figure 6:**
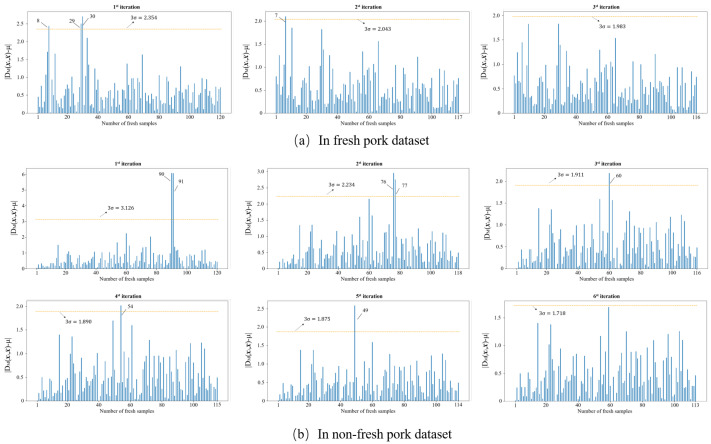
Iterative process of abnormal samples elimination.

**Figure 7:**
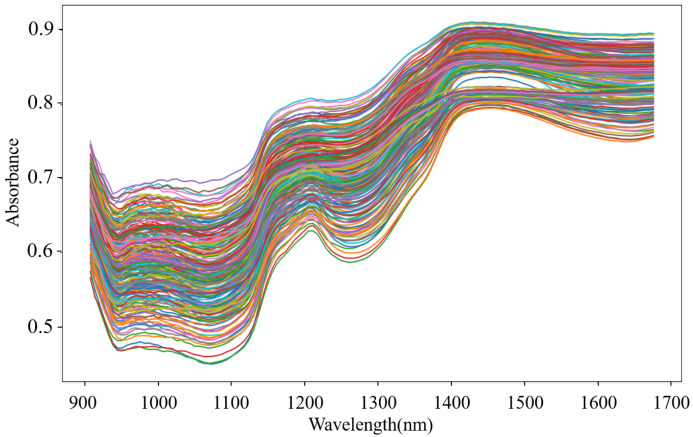
NIR spectra of samples after outlier detection.

Outliers that exist in the dataset seriously interfere with the model construction. Hence, it is necessary to identify and eliminate them prior to modeling. In this study, the MD-IC method was employed to exclude outliers, and the process of determining whether a sample is an outlier is shown in Figure 6a,b. The fresh and non-fresh pork datasets were analyzed separately. For the fresh pork dataset, 3σ is 2.354 and three samples were identified as outliers in the first iteration; then, 3σ was updated as 2.043 and one sample was excluded as an outlier in the second iteration; finally, the method was stopped in the third iteration as no outlier was found. Similarly, seven outliers were eliminated in the fresh pork dataset in total. The spectra of the remaining 229 samples after the outlier detection are shown in Figure 7. The dataset processed by the MD-IC was employed for the following model construction.

### 3.2. Comparison of Different Classification Models

To investigate the effectiveness of the proposed method, three conventional machine learning algorithms and two popular deep learning-based strategies were introduced as the comparison group. In this study, models were evaluated via nested cross-validation and the average indices (accuracy, precision, sensitivity, and specificity) across test sets were adopted as the performance evaluation indices.

#### 3.2.1. Compared with Conventional Algorithms

The proposed network was compared with traditional models, including SVM, RF, and PLS-DA. The experimental results are summarized in Table 2. This shows that the average accuracy of SVM without any preprocessing is 90.42%. After standardization (std), the Acc rises to 90.82%, which is the best result among conventional models. Unlike SVM, the performances of RF and PLS-DA remain unchanged after standardization. In addition, the smoothing (sm) reduces the Acc of SVM slightly but raises that of RF and PLS-DA significantly. The results indicate that the same preprocessing method is not effective for all models and even brings about information loss when applied inappropriately. To extract features from spectra, the PCA is utilized before constructing the SVM and RF models. However, it leads to a reduction in the performances of the two models. We suspect the potential reason is that, as a linear transformation, it is hard for PCA to extract effective features to represent the raw spectra. Similarly, the performance of PLS-DA that integrates a linear feature extraction method is inferior to that of SVM and RF when no preprocessing method is performed. Hence, it is time-consuming and laborious to select an optimal preprocessing method. From the Table 2, it can be seen that the 1D-SE-ResNet yields the best performance with an Acc of 93.72%, Sen of 90.77%, and Spe of 96.25%, respectively. The SVM combined with standardization achieves the best precision, but it is only slightly higher than that of the proposed model. The comparison results demonstrate that the proposed model is able to extract useful information through hierarchical structure and can be used as an end-to-end method to simplify the modeling process.

#### 3.2.2. Compared with Other Deep Learning Algorithms

Except for conventional algorithms, we also compare the proposed model with 1D-CNN and 1D-ResNet. The structure of 1D-CNN, which consists of one convolutional block and two fully connected layers, is similar to the CNN model designed in [29]. The architecture of 1D-ResNet is the same as with the 1D-SE-ResNet, but the latter has an extra SE block. It can be seen from Table 3 that the accuracy of 1D-CNN is 88.13%, which is not satisfactory, as the shallow structure can only extract low-level features. Owing to the deeper configuration and residual block, the 1D-ResNet yields a relatively high accuracy (90.69%). The proposed 1D-SE-ResNet provides the best performance with an accuracy of 93.72% and outperforms the other models in the precision, sensitvity, and specificity. The results demonstrate that the SE block can effectively adjust the weights of channels and can improve the model performance.

**Figure 8:**
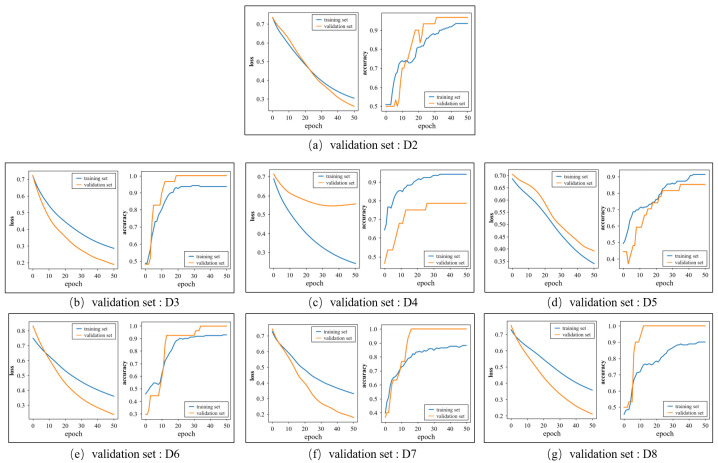
The change in the loss and accuracy over the training set and validation set in the training process.

Figure 8 shows the loss and accuracy curves of the training and validation sets when the group D1 is selected as the test set. Each subgraph corresponds to a fold in the inner loop and the label of corresponding validation set is marked below the subgraph. The accuracy curves of the training sets and validation sets all remain stable at the end of the training. In Figure 8a,b,e–g, the accuracy of the validation set is higher than that of the training set as the characters of the validation set are similar to the samples that were identified correctly in the training set. As for the loss curves, the loss values over the training set and validation set decline quickly and tend to converge, which denotes that the proposed model has a good ability to fit the dataset.

### 3.3. Ablation Study on Activation Function

As a nonlinear unit, the activation function is capable of dramatically promoting the representation capacity of the network. With the development of deep learning, considerable activation functions have been proposed in deep neural networks. In this study, we apply different activation functions in the 1D-SE-ResNet to compare their performances, including Sigmoid, ReLU, and ELU. Table 4 shows the classification results of 1D-SE-ResNet with different activation functions.

Sigmoid is a popular activation function in neural networks for its nice biological interpretations. However, it yields a less satisfactory performance than the other activation functions in this study. According to Equation (7), the input x is scaled to a value between 0 and 1. Additionally, the output σ(x) is saturated with the absolute value of x increasing, which may cause gradient disappearance during the back propagation. As the most frequently used activation function in deep learning, ReLU function achieves a relatively good performance with an accuracy of 91.97%. From Equation (8), it can be seen that the ReLU function directly outputs x if the input x is positive, which enables it to avoid the gradient disappearance problem. On the other hand, it outputs zero when the input is negative, which makes the network sparse bring about the “dead ReLU” issue. The ELU function (Equation (9)), a variant of ReLU, is similar to ReLU in the positive interval but adopts an exponential operation for the negative values, which avoids the dead neuron problem. In addition, the soft saturation characteristic makes ELU more robust to noise. By comparison, it can be seen that the 1D-SE-ResNet combined with ELU function achieves the best performance.

## 4. Conclusions and Discussion

This study presents a 1D-SE-ResNet classification model to identify pork freshness using the NIR spectra of pork samples. To improve the quality of dataset, the raw spectra have been processed by MD-IC method for outlier elimination. The training set, validation set, and test set are independent from each other as the dataset is split by the purchase date. Furthermore, the performance of the model is evaluated via the nested cross-validation, which ensures that all of the samples are tested and independent from the training set and validation set. Compared with traditional models such as SVM, RF, and PLS-DA, the proposed method does not involve tedious data preprocessing and achieves the best performance in terms of accuracy, sensitivity, and specificity, which indicates that the proposed method is able to simplify the modeling process as an end-to-end method. Moreover, a comparison between the proposed model and 1D-ResNet demonstrates that introducing a SE block improves the model performance significantly. This paper also evaluates the effects of different activation functions, and the results indicate that the ELU is the optimal one. In summary, this study provides an effective and promising approach for pork freshness detection based on NIRS.

However, it should be noted that more samples are needed for modeling in practical applications. In addition, the spatial information will be explored to improve the representational ability of the network and the pruning method will be further investigated to reduce the parameters of the model in consideration of the limited samples. The deep learning-based spectrum analysis methods are expected to be extended to various pork quality evaluation tasks.

## Figures and Tables

**Table 2:** Comparison between the 1D-SE-ResNet and conventional models in terms of average Accuracy (Acc), Precision (Pre), Sensitivity (Sen), and Specificity (Spe). The standardization and smoothing is denoted as std and sm, respectively.

Model	Preprocessing	Acc of Test Set (%)								Acc (%)	Pre (%)	Sen (%)	Spe (%)
		D1	D2	D3	D4	D5	D6	D7	D8				
SVM	/	92.86	96.67	79.31	78.57	100.0	92.59	90.00	93.33	90.42	94.86	86.03	95.00
	std	92.86	96.67	79.31	82.14	96.30	92.59	93.33	93.33	90.82	96.08	86.03	95.63
	sm	92.86	96.67	79.31	82.14	92.59	92.59	86.67	93.33	89.52	94.23	86.03	92.92
	PCA	92.86	96.67	79.31	75.00	100.0	92.59	90.00	93.33	89.97	93.75	86.03	94.17
RF	/	85.71	90.00	75.86	71.43	81.48	92.59	93.33	93.33	85.47	88.47	83.40	87.50
	std	85.71	90.00	75.86	71.43	81.48	92.59	93.33	93.33	85.47	88.47	83.40	87.50
	sm	85.71	93.33	75.86	75.00	85.19	96.30	100.0	93.33	88.09	92.03	85.06	90.83
	PCA	89.29	80.00	75.86	75.00	85.19	92.59	83.33	90.00	83.91	86.85	82.82	85.00
PLS-DA	/	96.43	53.33	79.31	67.86	66.67	100.0	83.33	86.67	79.20	81.11	86.03	71.46
	std	96.43	53.33	79.31	67.86	66.67	100.0	83.33	86.67	79.20	81.11	86.03	71.46
	sm	96.43	80.00	86.21	71.43	96.30	100.0	86.67	96.67	89.21	89.35	89.36	88.96
1D-SE-ResNet	/	89.29	96.67	100.0	78.57	85.19	100.0	100.0	100.0	93.72	96.06	90.77	96.25

**Table 3:** Comparison between the 1D-SE-ResNet and other two deep learning models.

Model	Acc (%)	Pre (%)	Sen (%)	Spe (%)
1D-CNN	88.13	90.19	87.82	88.13
1D-ResNet	90.69	91.34	89.36	92.08
1D-SE-ResNet	93.72	96.06	90.77	96.25

Acc, Pre, Sen, and Spe represent the average accuracy, precision, sensitivity, and specificity, respectively.

**Table 4:** Results of 1D-SE-ResNet with different activation functions.

Model	Activiation	Acc (%)	Pre (%)	Sen (%)	Spe (%)
1D-SE-ResNet	Sigmoid	81.00	80.55	75.07	89.17
	ReLU	91.97	94.53	89.10	94.58
	ELU	93.72	96.06	90.77	96.25

Acc, Pre, Sen, and Spe represent the average accuracy, precision, sensitivity, and specificity, respectively.

## Data Availability

Not applicable.
